# 11β-Hydroxysteroid dehydrogenases and the brain: From zero to hero, a decade of progress

**DOI:** 10.1016/j.yfrne.2010.12.001

**Published:** 2011-08

**Authors:** Caitlin S. Wyrwoll, Megan C. Holmes, Jonathan R. Seckl

**Affiliations:** Endocrinology Unit, Centre for Cardiovascular Science, Queen’s Medical Research Institute, The University of Edinburgh, Edinburgh EH16 4TJ, United Kingdom

**Keywords:** 11β-Hydroxysteroid dehydrogenase, Glucocorticoids, Mineralocorticoids, HPA axis, Ageing, Anxiety, Developmental programming

## Abstract

Glucocorticoids have profound effects on brain development and adult CNS function. Excess or insufficient glucocorticoids cause myriad abnormalities from development to ageing. The actions of glucocorticoids within cells are determined not only by blood steroid levels and target cell receptor density, but also by intracellular metabolism by 11β-hydroxysteroid dehydrogenases (11β-HSD). 11β-HSD1 regenerates active glucocorticoids from their inactive 11-keto derivatives and is widely expressed throughout the adult CNS. Elevated hippocampal and neocortical 11β-HSD1 is observed with ageing and causes cognitive decline; its deficiency prevents the emergence of cognitive defects with age. Conversely, 11β-HSD2 is a dehydrogenase, inactivating glucocorticoids. The major central effects of 11β-HSD2 occur in development, as expression of 11β-HSD2 is high in fetal brain and placenta. Deficient feto-placental 11β-HSD2 results in a life-long phenotype of anxiety and cardiometabolic disorders, consistent with early life glucocorticoid programming.

## 1 Introduction: An unhorrible history

1953 was a key year in biology: Crick and Watson discovered the structure of DNA, Howard and Pelc described the cell cycle and the Nobel Prize in Physiology or Medicine went to Hans Krebs for the eponymous tricarboxylic acid cycle. In the same year an arcane enzyme reaction catalysing glucocorticoid metabolism was discovered by Amelung and colleagues in Frankfurt. This occurred just 3 years after Kendall, Hench and Reichstein had won the Nobel Prize for the isolation of cortisone (‘compound E’) and shown its spectacular effects in treating patients with rheumatoid arthritis [96]. Amelung et al. [9] administered cortisone to rats and incubated cortisone with homogenates of various organs and found conversion to Kendall’s ‘compound F’ (cortisol). They localised the activity to microsomes and found the highest activity in liver with some also in kidney and muscle. This enzyme activity was 11β-hydroxysteroid dehydrogenase (11β-HSD). Until the late 1980s this reaction was considered arcane, one of a number of pathways of metabolism of glucocorticoids by liver and other organs, a topic of interest to steroid aficionados but of little main-stream biomedical concern.

A number of reports described deficiency in the inter-conversion of cortisol and cortisone in association with a very rare disease, the syndrome of “apparent mineralocorticoid excess” (AME). This condition was fatal in the few children reported [182,244,245,277], who presented with severe hypertension and blood biochemistry compatible with mineralocorticoid excess with sodium retention, potassium loss and metabolic alkalosis. Paradoxically, despite fully suppressed plasma renin activity, AME was accompanied by undetectable levels of all known mineralocorticoids, such as aldosterone and deoxycorticosterone. In the mid 1980s, Edwards and colleagues in Edinburgh investigated a unique patient with AME who had survived to adulthood [260]. In elegant clinical investigations they showed that the mineralocorticoid excess was due to cortisol. Normally, in humans and other mammals, cortisol has little or no mineralocorticoid activity *per se*. Nonetheless, in the adult AME patient, suppression of endogenous cortisol with the synthetic glucocorticoid dexamethasone reversed mineralocorticoid excess and concurrent re-administration of physiological doses of cortisol recapitulated mineralocorticoid excess, an effect not seen in healthy controls. The Edinburgh investigators also recognised that the syndrome was analogous to the effects of liquorice, long known to cause hypertension, and showed that ingestion of liquorice in humans produced AME only in the presence of cortisol [265].

In a scientific serendipity, Evans and his colleagues at the Salk Institute had just cloned the human mineralocorticoid receptor (MR) and were surprised to note that, *in vitro*, MR bound the physiological glucocorticoids cortisol and corticosterone and the mineralocorticoid aldosterone with similar affinity, a finding mooted from earlier studies using semi-purified receptor preparations [81] and functional studies in hippocampus [218]. It was then that the penny dropped and the Edinburgh group [68], as well as Funder and colleagues in Melbourne [72], recognised that selectivity of MR in the kidney *in vivo* was not due to any intrinsic specificity for aldosterone over glucocorticoids but to the activity of 11β-HSD. In the kidney, in the presence of 11β-HSD, cortisol was efficiently metabolised to inert cortisone, which does not bind to receptors. Only aldosterone, which is not a substrate for 11β-HSD, was able to gain access to otherwise non-selective MR. Mutations of the enzyme in AME sufferers or its inhibition by liquorice led cortisol ‘illicitly’ to bind and activate MR causing sodium retention, potassium loss and hypertension.

For the corticosteroid system this was the first example of pre-receptor metabolism gating steroid access to receptors. Pre-receptor activating systems had been shown for sex steroid receptors, with 5α-reductase type 2 converting the weaker androgen receptor agonist testosterone to more potent dihydrotestosterone in male secondary sexual structures [210,292] and aromatase converting androgens into estrogens in target tissues such as mammary gland and bone, thus providing ligand for estrogen receptors [203,237]. Analogous systems have also been described for thyroid hormone receptors with monodeiodinase isozymes inactivating or activating thyroid hormones in a cell-specific manner [80].

## 2 One enzyme or two?

**Fig. 1:**
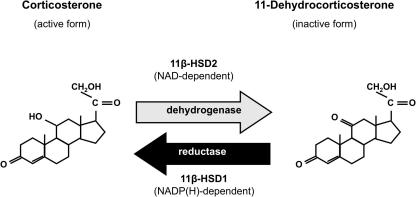
The enzymatic actions of 11β-hydroxysteroid dehydrogenase (11β-HSD) in inter-conversion of active and inactive glucocorticoids in rodents. Active glucocorticoid (corticosterone) is metabolised by 11β-HSD2 to its inactive form (11-dehydrocorticosterone) while regeneration can occur via 11β-HSD1.

An 11β-HSD activity had been purified and encoding cDNA clones isolated from rat liver by Carl Monder and colleagues in New York in the mid 1980s [1]. In tissue homogenates and microsomes this activity was bi-directional, containing both 11β-dehydrogenase (glucocorticoid inactivating) and 11β-reductase (glucocorticoid regenerating) activities, fuelled by NADP(H) as co-substrate, and had a modest affinity (high nM Km) for glucocorticoids (Fig. 1). This enzyme was expressed in rat kidney spawning suggestions that it underpinned MR selectivity and AME. However, a series of concerns (there are few MR in liver, the highest site of expression of this enzyme; the enzyme was expressed in the proximal tubule whereas MR are in the distal nephron; no mutations in the encoding gene were found in AME patients; the enzyme is bi-directional in homogenates yet apparently a unidirectional dehydrogenase in kidney *in vivo*) undermined the ‘one enzyme’ hypothesis. In 1993, Seckl and colleagues in Edinburgh [27] and Naray-Fejes-Toth and colleagues in Dartmouth, NH [231], isolated and characterised a novel enzyme from human placenta and rat kidney, respectively. This was distinct from the enzyme described by Monder, being a high affinity (low nM Km) exclusive 11β-dehydrogenase which used NAD rather than NADP(H) as co-substrate. In 1994, Krozowski’s group [6] isolated a cDNA encoding this ‘renal’ 11β-HSD from human kidney, White and colleagues found the same enzyme in sheep kidney [2], the rodent homologues were soon cloned [214] and an identical enzyme purified and its encoding cDNA cloned in the human placenta [27]. The new enzyme was called 11β-HSD type 2 to distinguish it from Monder’s 11β-HSD type 1 (Fig. 1). 11β-HSD2 is highly expressed in aldosterone-selective target tissues such as the distal nephron [226], colon [301], salivary glands [225] and skin [125], thus serving to confer aldosterone specificity on MR. Expression of 11β-HSD2 mRNA has also been localised to the adrenal gland [249], and vasculature [39,90], as well as in placenta and is widespread in the mid-gestational fetus [28]. Mutations in *HSD11B2* encoding 11β-HSD2 are found in patients with AME [65] and mice homozygous for targeted disruption of the *hsd11b2* gene faithfully recapitulate AME [134].

This left open the question of what Monder’s 11β-HSD1 enzyme, which was highly expressed in liver and also rat kidney was doing? Several groups suggested it might be a lower affinity 11β-dehydrogenase. In 1994, Seckl, Walker and their colleagues in Edinburgh showed that whilst bi-directional in homogenates, 11β-HSD1 acted as a predominant 11β-reductase in intact cells and *in vivo,* including in humans [113,155,215]. 11β-HSD1 is highly expressed in liver, adipose tissue, immune system cells and, in some species, in testes and ovary, with low-level expression widespread. In most tissues glucocorticoid regeneration is the preferred reaction although, unlike 11β-HSD2, its direction is dependent on levels of co-substrate. Indeed 11β-HSD1 is located inside the inner leaflet of the endoplasmic reticulum where its co-precipitates with hexose-6-phosphate dehydrogenase (H6PDH) which appears to be the major source of generation of NADPH driving 11β-reduction [66]. Deficiency or knock-out of H6PDH leads to reaction reversal of 11β-HSD1, though the importance of this remains uncertain under most physiological circumstances [143,144].

Here we review the biology of 11β-HSDs and focus on their role in determining glucocorticoid access to the developing and adult brain. We highlight their biology in health and role in the pathogenesis of disease through the lifespan.

## 3 11β-HSD and its place in the world of corticosteroid signalling in the brain

Glucocorticoids have profound effects on pre- and post-natal brain development. They are essential for normal maturation in most regions of the developing CNS, initiating terminal maturation, remodelling axons and dendrites, and affecting neuronal and glial cell survival [171]. Either inadequate or excessive glucocorticoid levels cause abnormalities in neuronal and glial structure and function that often impact throughout the lifespan. Similarly in adulthood, either excessive or deficient glucocorticoid action affects myriad brain functions, altering biochemistry, neurotransmission, cell structure, birth and death [118,120,160,234,235,285]. Thus accurate control of glucocorticoid levels and cellular action is critical for brain development and function. Whilst the precise mechanisms by which plasma glucocorticoid levels are regulated and thereby affect brain function is beyond the scope of this review, the classical view ascribes such regulation merely to the activity of the hypothalamic–pituitary–adrenal (HPA) axis, with its key forward drivers (stress, diurnal cues) and its well-described negative feedback control.

At the cellular level, the pervasive effects of glucocorticoids are largely a consequence of their transcriptional effects mediated via binding to high affinity MR and/or to the lower affinity glucocorticoid receptor (GR). Many genes, perhaps 5% of the genome, are glucocorticoid targets, albeit few if any exclusively so. Target genes include receptors, enzymes, neurotransmitters, calcium activation, ion channels, cytoskeleton, cellular transport, growth and metabolism [304].

Beyond plasma glucocorticoid levels and cellular GR and MR density, target tissue availability of glucocorticoids is also regulated in blood by their plasma protein binding, largely to corticosteroid-binding globulin (CBG) as well as albumin. CBG binds physiological glucocorticoids (cortisol, corticosterone) with high affinity, but has low or minimal affinity for their inert 11-keto-forms (cortisone, 11-dehydrocorticosterone) or for synthetic glucocorticoids (dexamethasone, prednisolone, triamcinolone) or mineralocorticoids (aldosterone, deoxycorticosterone, fludrocortisone) [26,229]. CBG may also act to deliver glucocorticoids to target cells. Binding to CBG and lower affinity proteins such as albumin ensures that only a small amount (2–5%) of physiological glucocorticoid is ‘free’ in the circulation [26,229]. However, CBG’s capacity to bind steroids can be flooded by high diurnal peak or stress levels of glucocorticoids when much becomes free. Severe illness/chronic stress often suppresses CBG production with a consequent increase in ‘free’ glucocorticoid, albeit with diminution of the delivery function of CBG [274]; the balance for glucocorticoid signalling is, as yet, poorly understood.

As glucocorticoids are highly lipophilic they readily diffuse across biological membranes into the cytoplasm, however, a role for membrane transporters is emerging. The Mdr/p-glycoprotein/ABCB1 transporter acts particularly at the blood–brain barrier (but also on other membranes) to partially exclude specific corticosteroids from brain (as in many peripheral organs), although such membrane ‘barriers’ are not absolute. Nonetheless p-glycoprotein minimises access of synthetic steroids like dexamethasone to the brain [169] and appears responsible for the preferential access of the non-substrate corticosterone rather than cortisol to human cerebrospinal fluid [124]. Nonetheless, the 10-fold molar excess of cortisol over corticosterone in human blood militates for its predominant role in hypothalamic–pituitary–adrenal (HPA) axis feedback [217]. Inward glucocorticoid carriers and pumps, mirroring the monocarboxylate transporter 8 which regulates thyroid hormone access to the brain and other organs [99], are being sought but remain, as yet, poorly defined.

Once inside the cell, corticosteroids bind to the two main types of intracytoplasmic receptors; GR and MR [71,165,220]. An additional nuclear receptor, the pregnane X receptor (PXR; known as SXR in humans) binds many synthetic glucocorticoids albeit with much lower affinity than GR and MR [129]. PXR is highly expressed in liver, but little if at all in brain parenchyma. However, PXR is present in CNS capillaries [18] where it directly up-regulates p-glycoprotein, perhaps forming a mechanism to attenuate brain exposure when plasma cortisol levels are chronically high. Additionally, MR and probably GR mediate rapid non-genomic effects probably via sites on the cell membrane [50]. The detailed biology of these important new actions is only beginning to emerge.

GR are widely if not ubiquitously expressed in neurons and glia. In contrast, high levels of MR are confined to hippocampus, septum and scattered nuclei in the brain stem [11]. However, many other regions have low-levels of MR and its role in signalling glucocorticoid actions in these sites is becoming clearer. Moreover, specific challenges may induce MR (and GR) in loci of otherwise low expression revealing novel functions such as neuroprotection in neocortex under cell challenges such as hypoxia and hypoglycaemia [136,157]. MR have a sub-nanomolar affinity (Kd ∼ 0.5 nM) for corticosterone and cortisol. When these glucocorticoids are not locally inactivated by 11β-HSD2, as in the adult hippocampus, MR are thought to be largely occupied at even the low-levels of ‘free’ glucocorticoids during the diurnal nadir [41,176,218]. Thus it is assumed hippocampal MR signalling is predominantly (not exclusively) determined by MR density. In contrast, GR have a lower (∼5 nM) Kd for physiological glucocorticoids and are barely occupied under basal levels of steroids, but become progressively activated as glucocorticoid levels rise during ultradian pulses, the diurnal maximum or a stress response [51,218,219,258].

Over and above all these factors, within cells, 11β-HSD acts as a major determinant of glucocorticoid access to receptors in peripheral tissues (reviewed in [65,239]). However, unlike the kidney and other classical aldosterone-selective target tissues, MR in the CNS largely bind physiological glucocorticoids *in vivo* [70], apart from discrete areas regulating blood pressure and salt appetite, reflecting the 100–1000-fold molar excess of glucocorticoids in the circulation. Thus the now ‘classical’ role of 11β-HSD2 in generating aldosterone-selective access to MR is minimal in the adult CNS. So is there any 11β-HSD in the brain and, if so, which isozyme(s)? Here we review this intriguing issue.

## 4 11β-HSD1

### 4.1 Historic studies

11β-HSD1 is the main isozyme found in the adult mammalian CNS. It was originally described in neuronal and glial cell lines in the 1960s. Using at that time cutting-edge histochemical and biochemical techniques, 11-keto oxidation of steroids was found in mouse, rat, dog and primate whole brain extracts, as well as fetal brain and the C6 glioma cell line [86,87,175,209,252]. Thereafter, inter-conversion of radiolabelled cortisol and cortisone *in vivo* and *in vitro* confirmed 11β-HSD activity in mouse brain, at lower levels than found in liver, kidney and placenta [33]. In contrast, the key studies in the late 1980s, which uncovered the crucial role of 11β-HSD in preventing glucocorticoids from binding to renal MR *in vivo* [68,72], did not find 11β-HSD activity in the hippocampus, data interpreted as demonstrating that the non-selectivity of hippocampal MR for corticosteroid ligands *in vivo* reflected the absence of 11β-HSD.

Subsequent re-examination of this issue, however, clearly demonstrated 11β-HSD activity in homogenates, first of rat cerebellum [179] and then in a broad range of rat CNS regions, including the hippocampus [139,180]. 11β-HSD activity is highest in the cerebellum, hippocampus and neocortex, with levels some 10–30% of those in kidney and liver [139,179]. 11β-HSD is also clearly detectable in most other brain subregions, including the hypothalamus, amygdala and brain stem [139,180,241]. The anterior pituitary also has high 11β-HSD activity [139,180]. Other mammalian species also express 11β-HSD activity in the CNS [115] including the post-mortem human brain [233]. Whilst there is some discordance in the earlier literature on the expression of 11β-HSD2 mRNA, and perhaps confusion generated by highly sensitive PCR-based methods which inevitably detect occasional transcripts, the vast majority of 11β-HSD mRNA and activity in the adult mammalian CNS is 11β-HSD type 1. The exception may be a few discrete nuclei in the hind brain/brain stem, notably the nucleus of the tractus solitarius (NTS), which expresses 11β-HSD2 mRNA in adult rodents.

## 5 Distribution of 11β-HSD1 in the CNS

### 5.1 11β-HSD1 in the adult brain

11β-HSD1 is widely distributed in the CNS, albeit with an uneven pattern of expression. The enzyme mRNA, protein and activity are found in neurons and in glia [180]. High adult expression is found in the cerebellum, hippocampus and cortex, with a curious patchy microdistribution, high in some cells, lower in others, that remains unexplained [180]. Higher levels are found in specific cells, for instance Purkinje cells of the cerebellum, CA3 pyramidal cells of the hippocampus and layer V neurons of the neocortex [180]. Lower expression is found in most cells of the CNS and spinal cord and includes notably the paraventricular nucleus of the hypothalamus, a key locus for glucocorticoid feedback control of the HPA axis [180]. 11β-HSD1 is also expressed in anterior pituitary cells including corticotrophs [131].

In general mRNA expression is paralleled by immunohistochemistry and by enzyme activity. The former has been hampered by the dearth of monospecific antisera, though western analysis suggests a single band in CNS at 34 kDa, the expected size of the full-length translated protein allowing for some glycosylation. However, most antisera reveal additional bands, not only dimers (68 kDa), but also alternative sizes that may or may not be products of the 11β-HSD1 gene.

At a subcellular level, work has similarly been hampered by a lack of highly selective antisera. In peripheral cells, 11β-HSD1 is located within the inner leaflet of the endoplasmic reticulum [201]. Early immunocytochemical studies suggested more widespread locations in neurons including on the cell membrane. Such data require confirmation and consequent speculation of a role for 11β-HSD1 in gating corticosteroid access to membrane MR [119], and perhaps GR, and thus modulation of rapid non-genomic effects, remains to be explored.

### 5.2 11β-HSD1 in the developing brain

Glucocorticoids play an important role during development, affecting the growth and differentiation of a number of tissues and organs, including the central nervous system [52,172]. High-dose glucocorticoid administration during the late prenatal and early post-natal period in rodents leads to permanent inhibition of brain growth, with reduced neurogenesis and glial proliferation, attenuated dendrite formation and behavioural and neuroendocrine impairments [23,49,85,283], often resulting in long term consequences on brain structure and function known as ‘programming’ (see section on developmental programming). Although it is 11β-HSD2 that is considered important in development, playing its part in maintaining a low glucocorticoid environment for the growing fetus (see section on 11β-HSD2), 11β-HSD1 also has an important role in late gestation. One mechanism to protect the fetus from very high levels of maternal glucocorticoids is to induce a period of stress hyporesponsiveness in the dam during pregnancy, which may occur, in part, from increased expression of 11β-HSD1 in the hypothalamus decreasing the forward drive on the HPA axis [121]. In rodents, 11β-HSD1 is also expressed in the placenta from E16, perhaps to boost the glucocorticoid surge near the end of gestation to ensure fetal maturation [34]. In the fetal brain, however, expression of 11β-HSD1 mRNA in the ovine fetal hippocampus is detectable at mid-gestation, rises until late gestation but decreases near to parturition and is not affected by prenatal glucocorticoid treatment [254]. In the rat [58,178] and mouse [257], 11β-HSD1 mRNA is not observed in the fetal brain until late gestation (>embryonal day (E) 16), a time when 11β-HSD2 is declining, and increases with age, although one report failed to detect 11β-HSD1 mRNA in fetal brain at all [273]. Treatment with dexamethasone in late gestation did increase 11β-HSD1 expression in the hippocampus of the newborn [294] as well as adult offspring [251], implicating it in the fetal programmed adult phenotype.

The activity in neonatal rat brain is likely to be 11β-reductase, which is the main reaction direction of 11β-HSD1 in primary (late) fetal hippocampal cell cultures [215]. Intriguingly, recent data indicate that 11β-HSD1 knock-out mouse (11β-HSD1^−/−^) pups are heavier at birth (controls: 1.344 ± 0.028 g; 11β-HSD1^−/−^: 1.468 ± 0.033 g, *P* < 0.05), suggesting a possible general role for 11β-HSD1 expression in cell maturation during late fetal and early post-natal life (D.J. Stenvers, J.R. Seckl and M.C. Holmes, unpublished observations) again introducing the potential for 11β-HSD1 and programming effects. This suggests that care should be taken in treating pregnant women with emerging selective 11β-HSD1 inhibitors.

## 6 Regulation of 11β-HSD1 expression

Given the importance of 11β-HSD in determining glucocorticoid action, many studies have addressed the regulation of enzyme activity. Dexamethasone, a synthetic glucocorticoid which is conventionally thought to be a poor substrate for 11β-HSDs, induces 11β-HSD1 gene expression and activity in rat hippocampus and liver and a variety of other peripheral cells [91,155,290]. Similar effects are found in other brain regions including the cortex, cerebellum and hypothalamus of the rat [155] and the hippocampus of the mouse (Teelucksingh, PhD Thesis, University of Edinburgh). However, care should be taken in interpreting these data as preliminary evidence using 19F-magnetic resonance spectroscopy of dexamethasone *in vivo* suggests that dexamethasone can be metabolised by 11β-HSD1 [193].

Glucocorticoid induction of cerebral 11β-HSD1 requires several days to become manifest. The mechanism is probably direct, as 11β-HSD1 induction by dexamethasone is also seen in primary hippocampal cells in culture [215]. Similar direct induction of 11β-HSD1 and its mRNA is observed in a variety of primary cells *in vitro* [31,44,69,91,113,269,300], although regulation *in vivo* is tissue-specific and considerably more complex [111,114,116,173,309]. Indeed, the cloned rat 11β-HSD1 gene proximal promoter region contains putative GRE half-sites [177] and promoter–reporter constructs indicate that the 11β-HSD1 promoter contains a functional glucocorticoid response element within 3700 base pairs of the transcription start site [286]. However, there is considerable evidence that glucocorticoid regulation of 11β-HSD1 is indirect. The *Hsd11b1* gene is transcribed from three promoters, P1-3 [30,177], but transcription in the brain, as well as liver and adipose tissue, is predominantly from P2 and is dependent upon C/EBPα and β [30,303]. Glucocorticoid regulation of human *HSD11B1* gene appears to be indirect and requires C/EBPβ binding to the P2 promoter, in skin and lung (C/EBPβ itself is up-regulated by glucocorticoids) [91,232]. Other transcription factors have been shown to regulate 11β-HSD1 transcription in the peripheral tissues by acting on the p2 promoter: PPARα [98], PPARγ [22], HNF1α [248], LXRα [268], but all act indirectly. More work is needed to determine 11β-HSD1 promoter regulation in the brain.

Arthritis stress for 15 days, which persistently and markedly elevates plasma corticosterone levels, also induces hippocampal 11β-HSD1 [155]. This is consistent with inflammatory stress being a major activator of 11β-HSD1, with proinflammatory cytokines increasing 11β-HSD1 expression [311]. This has prompted the notion that hippocampal 11β-HSD1 may function as an additional level of protection of vulnerable neurons from the endangering metabolic effects of chronically elevated glucocorticoid levels [155,181]. However, induction of 11β-HSD1 as a reductase would be predicted to *increase* cellular exposure to glucocorticoids and thus amplify any deleterious effects! In keeping with this, in the tree shrew, chronic psychosocial stress (for 28 days) attenuates hippocampal 11β-HSD activity [115]. Thus, (i) there are species differences, (ii) there is a complex time course of effects of glucocorticoids upon 11β-HSD1 expression or (iii) the effects of chronic inflammatory stress on hippocampal 11β-HSD1 differ from other chronic stimuli to the hypothalamic–pituitary–adrenal axis.

The case of inflammatory up-regulation of 11β-HSD1 in the brain, begs the question, which cells are showing the up-regulation, neurones or glia? It has been reported that microglia, the phagocytes of the brain, express 11β-HSD1. This is up-regulated when activated [84] as expected from the cells’ monocyte lineage. 11β-HSD1 in neurones, however, may be differentially regulated.

Other prominent regulatory factors of 11β-HSD in peripheral tissues include estrogen, growth hormone, thyroid hormones and insulin [91,145,146,153,154], but none of these have been shown to affect 11β-HSD1 in the CNS. Overall, regulation of 11β-HSD1 in the brain is inadequately understood.

## 7 Reaction direction, redox potential and hexose-6-phosphate dehydrogenase

**Fig. 2:**
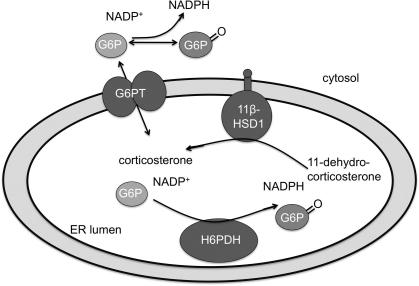
Orientation of 11β-HSD1 in the endoplasmic reticulum and its relationship with H6PDH. 11β-Hydroxysteroid dehydrogenase type 1 (11β-HSD1) is located on the luminal side of endoplasmic reticulum (ER) and the N-terminus is embedded into the membrane of the ER. The system comprising the glucose-6-phosphate (G6P) transporter and hexose-6-phosphate dehydrogenase (H6PDH) is crucial for transport of G6P to the H6PDH enzyme. G6P binds to the H6PDH to form 6-phospho-gluconolactone (G6P=O) resulting in generation of NADPH inside the lumen of the ER. The NADPH thus produced is utilised by 11β-HSD1 for the reduction of 11-dehydrocorticosterone to corticosterone.

The bi-directional capability of 11β-HSD1 suggests the same enzyme can increase or decrease intracellular glucocorticoid action depending on the context, particularly the cellular redox status. In contrast to bidirectionality in homogenates or purified enzyme preparations, in intact peripheral cells 11β-HSD1 usually acts as a predominant 11β-reductase, regenerating active glucocorticoids from inert 11-keto forms. For 11β-HSD1 to act as an efficient reductase it requires high levels of NADPH (an NADPH:NADP ratio >10). This gradient is thought to be generated by hexose-6-phosphate dehydrogenase (H6PDH) in the inner lumen of the endoplasmic reticulum (ER) [Fig. 2; [66]], where 11β-HSD1 associates with H6PDH through direct protein–protein interactions [14] to maximize efficiency [201]. Mutations in H6PDH in the mouse and human attenuate 11β-HSD1 oxido-reductase activity and reveal dehydrogenation [143,144], but does this matter in the brain?

As in peripheral tissues, 11β-HSD activity in homogenates of whole brain or CNS subregions is bi-directional. 11β-HSD activity in homogenates of brain subregions is markedly potentiated by addition of exogenous dinucleotide co-substrate *in vitro*, whereas in kidney or liver, activity is only marginally altered [86,139,179,180], perhaps reflecting lower levels of endogenous NADP(H) in the brain [82]. This has spawned the concept that variations in co-substrate levels may determine enzyme activity and direction in the brain *in vivo* [139,179,181]. Using immunocytochemistry, Gomez-Sanchez et al. [83] found patchy low expression of H6PDH in the brain that was not fully congruent with 11β-HSD1. Whilst this observation implies that 11β-HSD1 may act primarily as a dehydrogenase in the brain, this has not been observed. Indeed, in intact cells from hippocampus, cortex and cerebellum 11β-HSD1 acts as a near exclusive reductase [215]. Perhaps alternative sources of NADPH drive 11β-reductase in intact brain cells *in vitro* and *in vivo*. NADPH concentrations and H6PDH activity can be very sensitive to glucose concentrations depending on the cell type [66]. Glucose-6-phosphate (G6P), the substrate for H6PDH, is transported from the cytosol to the ER via the G6P transporter (G6PT; Fig. 2). G6PT deficiency in mice or humans decreases 11β-HSD1 reductase activity due to lack of substrate for G6PDH [291]. G6P is not only a substrate for H6PDH but is also converted to glucose by the enzyme glucose-6-phosphatase α (G6Pase), linking metabolic and glucocorticoid pathways. G6Pase deficiency causes glycogen storage disease type 1 (von Gierke’s disease) and an increase in hepatic 11β-HSD1 reductase activity [291] due to elevated availability of G6P for H6PDH. Indeed H6PDH is integrated in the pentose phosphate pathway to generate reducing equivalents in the form of NADPH, crucial for reductive biosynthesis within cells and necessary for provision of ribose-5-phosphate for synthesis of nucleotides and nucleic acids. However, the brain is not a prominent target in glycogen storage disease type 1 with damage correlating merely with hypoglycaemia, the major peripheral manifestation [170]. Clearly there is much to discover about the determinants driving 11β-reductase in brain cells.

The stability of 11β-reductase in brain homogenates is reported greater than in liver [137–139]. Why this should be the case is unclear, but may reflect lower proteolytic or other degradative processes in brain or a reaction direction driven by more than a sufficiency of co-substrate generators other than H6PDH. Subtle tissue-specific differences in 11β-HSD1 co-processing (e.g. glycosylation, which possibly affects reaction direction [3]) has also been advocated to underlie the stabilization of the 11β-reductase component in brain, though no direct data address this speculation.

## 8 Functions of 11β-HSD1 in the brain

### 8.1 Tools to study 11β-HSD1 function

Investigation of the possible function of 11β-HSD1 in CNS-derived and peripheral cells *in vitro* and in brain and other organs *in vivo,* initially exploited liquorice-based ‘natural’ inhibitors of 11β-HSDs. The root of the liquorice plant, *Glycyrrhiza glabra,* synthesises a number of triterpenoids based around glycyrrhizin; glycyrrhetinic acid is the most potent and inhibits 11β-HSDs at low nM Km in cell homogenates [20,264]. These compounds are now known to be non-specific, inhibiting both 11β-HSD1 and 2 and also affecting gap junctions and some related short-chain ketoreductases such as 15-hydroxyprostaglandin dehydrogenase, albeit with 2–4 logs lower affinity than 11β-HSDs [15,88,112].

More recently, a number of genetically-manipulated mouse models have been employed, including transgenic over-expression and knock-out lines. Whilst redundancy and compensatory developmental effects complicate many such approaches, the derived data in the case of 11β-HSD1 are strengthened by the lack of redundancy since adult 11β-HDSD1^−/−^ mice cannot regenerate active glucocorticoids from inert 11-keto forms [135]. Moreover, unwanted developmental effects are minimised by the low endogenous expression of 11β-HSD1 in the fetus until near birth. Nonetheless, 11β-HSD1 is expressed in late fetal development and contributes to amplifying glucocorticoid signalling at least in the lung at term [110], and is clearly expressed postnatally [179,180] so developmental effects may contribute somewhat to knock-out phenotypes.

Recently, a number of selective 11β-HSD1 inhibitors have been reported. The first, arylsulphonamidothiazoles, inhibit 11β-HSD1 *in vitro* and *in vivo* and show >200-fold selectivity over 11β-HSD2 [4,16]. These agents lower plasma glucose and insulin in hyperglycaemic mice, reduce hepatic glucose production, decrease cholesterol, free fatty acids and triglyceride levels [5], recapitulating the 11β-HSD1^−/−^ mouse phenotype which represents lowered intracellular glucocorticoid action predominantly in liver and adipose tissue [186]. Similar effects are shown by other compounds, adamantyl triazoles, octyltriazoles, phenyl triazoles [13,97,323,324]. However, to date central effects of these 11β-HSD1 inhibitors have not been reported. This may reflect the therapeutic target prioritized by the pharmaceutical companies (and hence central effects were not monitored) or the difficulty of designing selective compounds passing the blood–brain barrier. However recent data suggest peripherally administered selective 11β-HSD1 inhibitors can target the enzyme effectively in the brain [256].

### 8.2 Effects in CNS cells

Whilst early studies of 11β-HSD in the CNS showed the presence of the enzyme, ideas of function were dominated by the spectacular biology of 11β-dehydrogenase in the kidney which was initially thought to be the same enzyme. With the discovery that 11β-HSD1 predominates in the adult mammalian CNS and is an 11β-reductase in intact clonal and primary cultures of liver and other cells, this interpretation was challenged [31,113,155]. Rajan and colleagues [215] showed that primary cultures of (fetal) hippocampal cells expressed 11β-HSD1 but not 11β-HSD2. The activity was exclusively an 11β-reductase and could be potently inhibited (Ki low nM) by carbenoxolone, the hemisuccinate (to promote absorption) derivative of glycyrrhetinic acid. *In vitro*, pre-treatment with glucocorticoids promotes hippocampal cell death in the presence of high but sub-lethal doses of excitatory amino acid glutamatergic neurotransmitters such as kainic acid [215]. Whilst intrinsically inert 11-dehydrocorticosterone is equipotent with active corticosterone in potentiating kainate neurotoxicity, addition of carbenoxolone, itself without neurotoxic effects, attenuates the toxicity of 11-dehydrocorticosterone, but not corticosterone, in hippocampal cell cultures [215]. These data support the 11β-reductase reaction direction of 11β-HSD in hippocampal cells and imply a potential role in amplifying intracellular glucocorticoid action. However, such *in vitro* studies cannot do more than indicate any *in vivo* importance.

### 8.3 Alternative reactions

11β-HSD1 has recently been reported to additionally catalyze inter-conversion of 7-position modified sterol and steroid substrates including the oxysterols 7-ketocholesterol to 7β-hydroxycholesterol [223,238]. This probably reflects the mirror-image structures involved; inverting 7-position modified ketosterol/ketosteroid rings produces a close resemblance to the known 11-keto-steroid substrates. Indeed, 11β-HSD1-dependent glucocorticoid conversion may be attenuated by competition from the alternative 7-oxysterol substrates [293]. The importance of such reactions in the brain is unexplored, but oxysterols such as 7-ketocholesterol may be neurotoxic and their levels rise with excitotoxicity and perhaps other pathologies [59,127]. The role of 11β-HSD1 and whether or not 7-keto and 7β-hydoxy cholesterol forms differ in these or other properties in the CNS remains uncertain. Additionally, 7-keto- and 7β-hydroxy derivatives of the neurosteroids dehydroepiandrosterone (DHEA) and pregnenolone may be metabolised by 11β-HSD1 [194]. 7-position modification of DHEA and pregnenolone may potentiate neurosteroid activity, for instance in cognitive enhancement with ageing [313], but any functional importance of 11β-HSD1 in these reactions has yet to be addressed.

### 8.4 11β-HSD1 and the HPA axis

Inter-individual differences in HPA axis underlie differential vulnerability to neuropsychiatric and metabolic disorders, although the basis of this variation is poorly understood. A major stress to one individual may underpin anxiety or depressive symptoms with chronically elevated glucocorticoids, while another may develop post-traumatic stress disorder (PTSD) associated with a tendency towards lower circulating glucocorticoids, or fail to elicit any lasting behavioural or neuroendocrine abnormality at all. Although the relationship between the different HPA axis states to the pathophysiology of these disorders is unclear, perhaps the most robust biological effect in psychiatry is altered glucocorticoid feedback efficacy upon the HPA axis in various disease states, notably blunted feedback in melancholic depression and enhanced feedback in PTSD [45,317]. Moreover, numerous reports of efficacy of glucocorticoid-lowering therapies in metabolic syndrome and depression [109,263,295,320] suggest a role in pathogenesis and/or maintenance of pathologic vulnerability. Clearly the genetic and developmental mechanisms that underpin individual differences in HPA axis function are of considerable importance.

Expression of 11β-HSD1 in sites within the brain that are responsible to the negative feedback actions of glucocorticoids (cerebral cortex, hippocampus, hypothalamus and pituitary), suggest this enzyme may be a key regulator of the HPA axis. Indeed, mice lacking 11β-HSD1 exhibited signs of attenuated glucocorticoid negative feedback, consistent with reduced glucocorticoid signalling within the brain [94]. Moreover, the mice had elevated nadir levels of plasma corticosterone, an exaggerated corticosteroid response to an acute stressor and the adrenal glands were enlarged [94]. Interestingly, when the 11β-HSD deletion was bred onto another genetic strain background (129/MF1 moved to C57Bl/6 J) the consequences of the deletion on HPA axis activity was considerably altered. In C57Bl/6 J mice, 11β-HSD1 deletion results in normal basal plasma corticosterone and an efficient negative feedback signal onto the brain, due to a compensatory rise in the levels of GR expression in the hippocampus and PVN of the hypothalamus [38]. Indeed the elevation of basal corticosterone appears to track with the 129 genotype. However, all 11β-HSD1 null mice have larger adrenals often with an exaggerated glucocorticoid response to stress [38]. Thus, although 11β-HSD1 appears to contribute to regulation of the HPA axis, the genetic background is crucial in governing the response to its loss. Similar variations in plasticity may underpin inter-individual differences in vulnerability to disorders associated with HPA axis dysregulation. While these data indicate that 11β-HSD1 inhibition does not inevitably activate the HPA axis beyond ‘compensatory’ elevation of ACTH to maintain plasma glucocorticoids, it does suggest that certain individuals treated with inhibitors could potentially have chronically increased cortisol levels. However, to date, trials of 11β-HSD1 inhibitors in rodent models or clinical trials have failed to uncover cortisol/corticosterone changes [228]. Note that the adrenocortical enlargement is considered ‘compensatory’ in as far as peripheral 11β-HSD1 contributes substantially (20–40%) to total daily glucocorticoid production by regenerating cortisol from inert cortisone largely in the liver and splanchnic bed [17,288]. Merely to replace this, the HPA axis must drive the adrenals to produce extra glucocorticoids. Use of a selective 11β-HSD1 inhibitor in humans increases serum levels of ACTH and ACTH-sensitive adrenal products such as dehydroepiandrosterone, but without changes in cortisol, and is presumed to reflect this process [228]. Whether or not human equivalent of the 129 mouse, with 11β-HSD1 deficiency-induced plasma glucocorticoid excess, will be found remains an important if unanswered question.

Tissue-specific alteration of 11β-HSD1 has added to our understanding of the role of this enzyme in regulating HPA activity and circulating glucocorticoid levels. If 11β-HSD1 is replaced only in the liver, using an ApoE-HSD1 transgene in 11β-HSD1^−/−^ mice from a strain that shows elevated circulating levels of corticosterone, the basal and stressed plasma corticosterone levels and the adrenal weights are normalised [205], implying peripheral 11β-HSD1 is sufficient to rescue HPA abnormalities seen in 11β-HSD1^−/−^ mice. Overexpression of 11β-HSD1 in the liver (i.e. on a wild type background) has no observable effect on circulating levels of corticosterone [206], nor does overexpression in fat [164] or the brain [102]. Furthermore, ectopic expression of the dehydrogenase 11β-HSD2 in fat, which reduces local glucocorticoid exposure, also has no effect on circulating glucocorticoid levels [126]. However, it still remains to be tested whether deletion of 11β-HSD1 solely in the brain is sufficient to recapitulate the HPA effects observed in a global knock-out on a ‘HPA-dysfunction susceptible’ strain background.

### 8.5 Circadian regulation

**Fig. 3:**
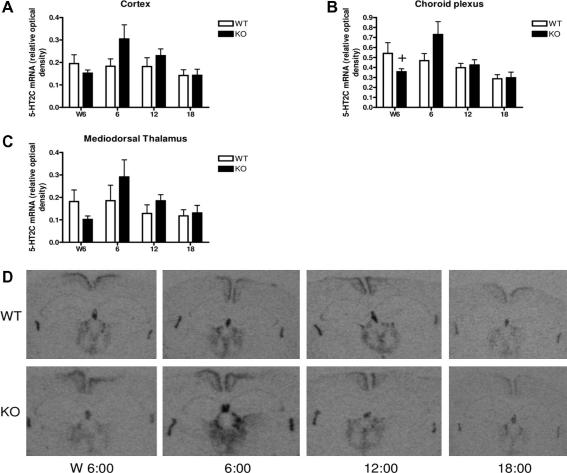
The effects of diurnal rhythm and voluntary exercise on 5-HT 2CR mRNA expression in 11β-HSD1^−/−^ (KO) and wild type mice (WT). Animals were kept in a constant light-dark cycle with lights on at 06:00 and lights off at 18:00. Mice were sacrificed at 06:00 (6), 12:00 (12), 18:00 (18), or after two months of voluntary wheel-running at 06:00 (W6). 5-HT 2CR mRNA expression was detected by *in situ* hybridisation histochemistry. Diurnal variation was seen in 11β-HSD1^−/−^, but not in wild type animals in (A) retrosplenial cortex (KO: *P* = 0.022, WT: *P* = 0.698) and (B) choroids plexus (KO: *P* = 0.001, WT: *P* = 0.232). (C) In the mediodorsal thalamus a tendency towards diurnal variation was observed in 11β-HSD1^−/−^, but not in wild type animals (KO: *P* = 0.090, WT: *P* = 0.596). (D) Representative photomicrographs. *n* = 4–6 for each condition. Values are mean ± SEM. ^+^*P* < 0.05 compared to corresponding 06:00 value.

Intriguingly, an abnormal circadian profile of plasma corticosterone levels was reported in 11β-HSD1 knock-out mice on a 129/MF1 background [94], suggestive of abnormalities in circadian signalling onto the HPA axis in the absence of 11β-HSD1. However, the altered circadian rhythm in plasma corticosterone and ACTH was not apparent in 11β-HSD1^−/−^ mice on the C57Bl/6 J strain background. Furthermore, the clock genes, Per1 and Per2, show normal circadian variation of expression in 11β-HSD1^−/−^ mice, but expression of the 5-HT_2C_ receptor, previously shown to be expressed in a circadian manner in the rat hippocampus where it is regulated by glucocorticoids [103], only showed circadian variation in 11β-HSD1^−/−^ mice, not in wild type controls (C57Bl/6 J; Fig. 3) suggesting this rhythm only becomes manifest when intraneuronal glucocorticoid levels are low in this species. Consistent with normal circadian patterns of gene expression and hormone levels, circadian wheel-running behaviour is unaltered in 11β-HSD1^−/−^ mice.

**Fig. 4:**
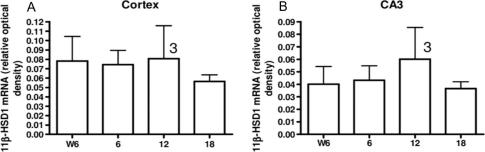
The effects of diurnal rhythm and voluntary exercise on 11-HSD1 mRNA expression in the cortex and hippocampus of wild type mice (C57BL/6J). Animals were kept in a constant light-dark cycle with lights on at 06:00 and lights off at 18:00. Mice were sacrificed at 06:00 (6), 12:00 (12), 18:00 (18), or after two months of voluntary wheel-running at 06:00 (W6). Gene expression was detected by *in situ* hybridisation histochemistry. 11-HSD1 mRNA expression in (A) cortex and (B) CA3 of wild type animals. *n* = 4–6 for each condition, except where a number above the bar indicates the n for that condition. Values are mean ± SEM.

Given the regulation of 11β-HSD1 by glucocorticoids, circadian changes in the enzyme in brain has been explored. In C57Bl/6 J mice there is no circadian variation in 11β-HSD1 mRNA in the hippocampus (Fig. 4; D.J. Stenvers, J.R. Seckl, M.C. Holmes, unpublished observations. Similarly in mice with diet–induced obesity, no rhythm in hippocampal 11β-HSD1 activity was observed [284]. However, 11β-HSD1 mRNA shows diurnal variation in lean, but not obese, Zucker rats [32]. Overall the impact of 11β-HSD1 on circadian regulation and of diurnal cues on brain 11β-HSD1 appear strain, species and state-dependent. This may have importance as 11β-HSD1 inhibitors may provide greater metabolic efficacy when given in the evening [284] in rodents, the time of the diurnal peak of glucocorticoids. It may have been postulated that an inhibitor would have minimal impact on intracellular glucocorticoid levels at the active glucocorticoid zenith, but this also coincides with the maximum for plasma levels of the 11-keto substrate for 11β-HSD1. Any impact of this in humans remains unexplored.

### 8.6 11β-HSD1 and appetite regulation

Overexpression of 11β-HSD1 in adipose tissue causes hyperphagia, whereas ectopic expression of 11β-HSD2 in adipose tissue induces hypophagia suggesting that glucocorticoid action within fat tissues controls appetite [126,164]. However, the 11β-HSD1^−/−^ mouse paradoxically shows increased appetite for high fat diet, at least for several weeks [184,185]. This suggests distinct, perhaps central effects of enzyme deficiency on appetite for calorie dense diets. Indeed, 11β-HSD1 (mRNA and enzyme activity) is expressed in the hypothalamic arcuate nucleus, a key locus for appetite control [180]. Intriguingly, 11β-HSD1 is induced in the arcuate nucleus by high fat feeding [57]. 11β-HSD1 null mice have altered neuropeptide gene expression in the arcuate, notably reduced anorexigenic cocaine and amphetamine-regulated transcript and melanocortin-4 receptor mRNAs suggesting increased ‘appetitive tone’ [57]. Importantly, under high fat diet challenge, the 11β-HSD1 null arcuate nucleus up-regulates orexigenic agouti-related peptide (AGRP) mRNA, whereas controls fed this obesogenic diet reduce AGRP expression [57]. The mechanisms appear to operate via μ-opioid receptor tone and imply that local 11β-HSD1 plays a role in central adaptive restraint mechanisms to dietary challenges.

### 8.7 11β-HSD1 and affective behaviour

In a significant proportion of patients suffering from depression, there is elevated cortisol production over 24 h, notably a rise in nadir plasma cortisol levels and a decreased amplitude of the circadian profile [37]. It was therefore suggested that elevated glucocorticoid signalling within the brain may play a role in the aetiology of depression, which is supported by the high incidence of depression in patients suffering from Cushing’s syndrome [40]. However, a consequence of high glucocorticoid levels is some down-regulation of GR in the brain and periphery, manifest in depression with reduced negative feedback as observed in the dexamethasone suppression test. The glucocorticoid hypothesis of depression therefore had been modified, as the behavioural abnormalities of mood are perhaps as likely due to low rather than high glucocorticoid signalling, a hypothesis supported by the affective phenotype of mice lacking GR selectively in the forebrain [24]. Hence, as 11β-HSD1^−/−^ mice have lower levels of corticosterone in the brain, they have been assumed to be susceptible to increased anxiety or depressive-like behaviours. Generally this was not found to be the case, at least on the C57Bl/6 background, in either elevated-plus maze or open field tests [312]. These findings are consistent with results from GR^+/−^ mice, which also have reduced (as opposed to abrogated) CNS glucocorticoid signalling but also do not show altered affective behaviours in the basal state [221]. Young and old mice with modest overexpression of 11β-HSD1 in the forebrain also showed no signs of anxiety, and do not exhibit altered GR or MR density either (at least in hippocampus), suggesting that low/normal to somewhat elevated glucocorticoid signalling does not increase anxiety or depressive-like behaviours [102]. Perhaps a ‘second hit’ such as altered monoaminergic neurotransmission is required to manifest affective impacts with modest changes of central glucocorticoid signalling?

### 8.8 Cognition in young animals

**Fig. 5:**
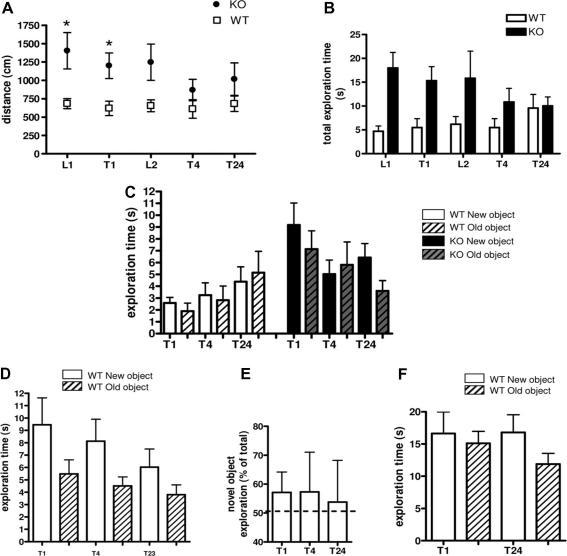
Behaviour of 11β-HSD1^−/−^ (KO) and wild type (WT) mice in the object recognition test. Two identical objects were presented to the mice in a learning session (L). In test sessions after 1 (T 1), 4 (T 4) or 24 (T 24) hours, the same (old) object and a new object were shown to the animals. (A) Distance travelled by 11β-HSD1^−/−^ and wild type animals (*n* = 12 per genotype) in experiment 1. ANOVA reveals a significant effect of genotype (*P* = 0.017) and a decrease in activity over sessions in 11-HSD1^−/−^ animals only (*P* = 0.022). (B and C) Exploration time of 11β-HSD1^−/−^ and wild type mice (*n* = 12 per genotype) in experiment 1. (B) 11β-HSD1^−/−^ mice show increased total exploration time compared to wild types, confirmed by ANOVA (genotype effect, *P* = 0.019). (C) No significant preference for the novel object over the old object was seen in either genotype. ANOVA reveals a significant session:genotype interaction effect (*P* = 0.010). (D and E) Exploratory behaviour of naïve wild type mice (*n* = 12) in experiment 2. Significant preference for the novel object was seen (D) in terms of absolute exploratory behaviour, confirmed by ANOVA (*P* = 0.005), but not (E) in terms of the percentage of total exploration time spent exploring the novel object. Dashed line indicates chance level. (F) Exploration time of naïve wild type mice (*n* = 15) in experiment 3. Values are mean ± SEM. ^*^P < 0.05 compared to corresponding wild type animals.

11β-HSD1 is widely expressed in hippocampus and neocortex suggesting its potential involvement in such processes as memory and learning. Young adult 11β-HSD1 null mice generally show normal performance in tests of cognitive function; for example normal acquisition and retention of spatial memory in the watermaze and Y-maze [312,315]. Whilst 11β-HSD1^−/−^ mice have impaired performance in the object recognition test, which examines memory and exploratory behaviour in a novel environment (Fig. 5) this was associated with hyperactivity which confounds assessment (D.J. Stenvers, J.R. Seckl and M.C. Holmes, unpublished observations). 11β-HSD1 null mice also unexpectedly show reduced memory retention after 24 h in the passive avoidance test (latency 16 ± 2.3 s vs. 63 ± 22.2 s). Reduced hippocampal MR signalling during the circadian trough, potentially a consequence of reduced glucocorticoid regeneration within MR-expressing hippocampal cells when basal corticosterone levels are low (as in young animals) and thus MR signalling might be modulated, is a plausible cause of the novelty-induced hyperactivity, and possibly also of the reduced associative memory in 11β-HSD1^−/−^ mice. However, the major cognitive phenotypes associated with 11β-HSD1 manipulations only emerge with ageing.

## 9 11β-HSD1 and the ageing brain

Chronic elevation of glucocorticoids is associated with affective, cognitive and even psychotic disorders [272]. Accumulating evidence suggests that cognitive impairments with ageing associate with elevated glucocorticoid levels in rodents and humans [168]. Indeed, maintenance of low glucocorticoid level throughout life, either via neonatal ‘programming’ of GR and MR in hippocampus which afford tighter HPA axis control, by antidepressant drugs which up-regulate GR and MR in the hippocampus and other feedback sites in adulthood to similar effect, or by adrenalectomy with low-dose glucocorticoid replacement in mid-life, prevent the emergence of cognitive deficits with age [140,166,314].

Aged 11β-HSD1^−/−^ mice resist the cognitive impairments seen in aged wild type mice. This occurs in various cognitive tasks of spatial memory, such as the watermaze [315] and the Y-maze [312]. Indeed the Y-maze test is sensitive enough to determine cognitive decline in mid-aged animals and again 11β-HSD1^−/−^ mice are protected from the deficits seen in congenic wild type animals. Importantly, the effects appear to be mediated upon cognitive processes, rather than confounders such as affective behaviour or locomotion which are broadly unchanged by 11β-HSD1 deficiency (although aspects of locomotion at least are clearly impacted by ageing *per se* [312]). Cognitive effects of 11β-HSD1 deficiency have been observed using two distinct in-bred genetic backgrounds (129, C57Bl/6 J) suggesting they may be generalisable.

Deficiency or inhibition of 11β-HSD1 in liver and adipose tissue causes local insulin sensitisation attenuating glucocorticoid driven processes such as hepatic gluconeogenesis and lipid β-oxidation. The effect is to reduce fasting and post-prandial glucose and insulin levels, triglycerides and atherogenic LDL-cholesterol, notably in obese animals and subjects with type 2 diabetes [135,184,185,228,289]. Type 2 diabetes is a risk factor for cognitive impairments with ageing [12,267] and cognition correlates with HbA1c, a marker of long-term glycaemic control, in non-diabetic elderly subjects [158]. However, several lines of evidence suggest that direct effects of 11β-HSD1 deficiency in the brain play a role.

First, whilst on the original 129 strain background, 11β-HSD1 deficiency was associated with modestly elevated plasma corticosterone levels [94], this is not seen on other strain backgrounds [312] and would under other circumstances anyway be anticipated to cause cognitive deficits not protection over the lifespan. Moreover, 11β-HSD1^−/−^ mice show strikingly reduced intrahippocampal corticosterone levels in the face of maintained or even elevated plasma glucocorticoids [312]. This illustrates the importance of the enzyme in determining effective intracellular glucocorticoid signalling within brain cells.

Second, though 11β-HSD1 deficiency certainly attenuates metabolic disease with dietary challenge, obesity or stress, there is little effect under basal conditions. The cognitive protection seen in aged 11β-HSD1^−/−^ mice under low stress housing and on normal chow diet was not associated with any alterations in body weight, plasma glucose or lipids, though plasma insulin levels were reduced [135,185]. It seems unlikely that modest lowering of insulin levels is alone responsible for cognitive improvements. However, the role of insulin signalling and processing in the CNS clearly merits further dissection, notably as the insulin degrading enzyme (IDE) is also important in beta-amyloid processing. Nor is this a generalised retardation of ageing since longevity appears unaltered in 11β-HSD1 deficient mice.

Third, 11β-HSD1 deficiency has effects upon the brain. Most notable is the effect upon long-term potentiation (LTP) in the CA1 region of hippocampal slices. LTP is a measure of synaptic plasticity and is associated with learning and memory function. Aged C57Bl/6 J control mice show attenuated LTP formation to a tetanic stimulus compared with young animals. In contrast aged 11β-HSD1^−/−^ mouse slices exhibit substantially greater LTP formation than similarly aged wild type preparations [312]. Again, whilst it is not possible to prove in such a model that the electrophysiological effects in hippocampal slices are due to the direct actions of 11β-HSD1 in the brain, this is a plausible scenario.

Fourth, glucocorticoids inhibit neurogenesis, a key process in the dentate gyrus which underpins its plasticity and could impact upon cognitive functions. Young 11β-HSD1^−/−^ mice show substantially increased hippocampal neurogenesis [312], as anticipated from the known inhibitory effects of glucocorticoids on the process. Aged mice have very little neurogenesis anyway in the dentate gyrus, and the presence or absence of 11β-HSD1 appears to make little difference to this [312]. Of course, greater neurogenesis in young animals might underpin a systematic ‘gain-of-function’ state. Whilst no cognitively enhanced functions have been reported in young 11β-HSD1^−/−^ mice, the tests employed may not be sensitive enough to discriminate such effects.

However, other data suggest that changes in 11β-HSD1 impact more with ageing. Thus transgenic mice engineered to over-express 11β-HSD1 in the forebrain (50% increase across the hippocampus) under the CAMIIK promoter, which is expressed only from the third post-natal week thus avoiding ‘developmental’ effects, show normal learning in the watermaze as mature (9 month old) adults, but subsequently develop memory impairments at 18 months of age (mid-life) using watermaze test of spatial learning and conditioned avoidance learning tests [102]. Whether or not these animals show altered neurogenesis remains to be explored, but the implication is that ageing *per se* is required for the effects of 11β-HSD1 manipulations to impact noticeably on cognitive function and that elevated 11β-HSD1 activity is sufficient to produce cognitive decline with ageing. This, together with the clear cognitive decline associated with up-regulation of endogenous 11β-HSD1 in the brain of C57Bl/6 mice in layer V of the cerebral cortex and CA3 regions of the hippocampus as they age [102], affords strong support for the notion that it is the enzyme in the brain that is crucial and perhaps causal to the cognitive impacts seen. It has even been suggested that cognitive decline with ageing is caused by a “Cushing’s disease of the brain”, paralleling the emerging biology of raised 11β-HSD1 in adipose tissue in human and monogenic rodent obesity [151,216] that causes metabolic disease [163,164]. This may sound all very intractable, but indeed even short-term (2 weeks) treatment with a CNS-active selective 11β-HSD1 inhibitor improves cognitive function in aged mice, at least in the low stress Y-maze task [256]. Thus the 11β-HSD1-associated part of the glucocorticoid contribution to cognitive ageing appears not entirely irreversible.

A further issue arises; what drives increased 11β-HSD1 in the brain with ageing? The limited data imply the age-related rise in glucocorticoid levels may further amplify their deleterious effects in the CNS by inducing 11β-HSD1 expression. An intriguing alternative is developmental programming, a process largely addressed below. Data in rodents suggest that prenatal exposure to excess glucocorticoids ‘programmes’ long-lasting increases in 11β-HSD1 transcripts in the hippocampus [251]. Any human relevance of such biology remains to be explored, but late-gestational programming of increased 11β-HSD1 in peripheral tissues occurs in non-human primates [196] so the extrapolation is perhaps not entirely absurd.

### 9.1 Human brain studies

*In situ* hybridization studies in post-mortem human brain have confirmed the expression of 11β-HSD1 in hippocampus, prefrontal cortex and cerebellum [233]. In two small, randomized, double-blind, placebo-controlled, crossover studies, carbenoxolone improved verbal fluency in healthy elderly men and verbal memory in patients with diabetes type 2. Similar finding have been reported in rodents [315].

In a prospective study in 41 healthy elderly men, the total body 11β-HSD1 ratio, but not other indices of glucocorticoid production or metabolism, predicted the decline in ventricular volume and cognitive function (processing speed) over the next 6 years [159], explaining 10% and 30% of the variance, respectively. These data suggest that elevated 11β-HSD1 may be predictive and even causal of cognitive decline and brain matter loss with ageing in humans. However, the location(s) of the 11β-HSD1 responsible for these effects is uncertain. Moreover, a potential link between cognition and metabolism should be highlighted. Since 11β-HSD1^−/−^ mice are insulin sensitized and have an atheroprotective lipid profile, it might be anticipated that the neuroprotective effect of the enzyme inhibitors could be secondary to metabolic and vascular effects. Chronic hyperglycemia in type 2 diabetes indeed associates with mild cognitive impairments [267]. Polymorphisms in *HSD11B1* gene have been linked to diabetes type 2 and hypertension, at least in Native Americans, and a rare polymorphism (rs846911-C/A) has been correlated with an increased risk of Alzheimer’s disease [54]. However, in a study of 194 participants of the Scottish Mental Survey the common variants did not associate with cognitive impairment with ageing and the rare polymorphism was not detected [56]. Nevertheless, although carbenoxolone enhances insulin sensitivity in healthy young volunteers [289] and patients with diabetes type 2 [10], in the elderly cognition studies there were no effects on indices of glycaemic control or serum lipids. The potentially synergistic effects of 11β-HSD1 inhibition on the brain and metabolism appear propitious, but the locus of action of selective 11β-HSD1 inhibitors needs to be defined. Nonetheless, 11β-HSD1 inhibition is an intriguing prospective therapy for cognitive decline with ageing, especially as even short-term use of CNS-active selective inhibitors improve memory function in aged rodents [256] as does non-selective carbenoxolone in elderly humans [233]. Any efficacy of such agents in more catastrophic pathological states of cognitive decline, such as Alzheimer’s disease, remains to be explored.

## 10 11β-HSD2

Within the cell, 11β-HSD2 is localised to the endoplasmic reticulum, with a cytosol-facing active site and co-factor binding domain, and binds to its substrate with around 100-fold more affinity than 11β-HSD1, suggesting it may play a more dominant role in corticosteroid metabolism in tissues if the two enzymes are co-expressed [65]. Importantly, 11β-HSD2 is not always colocalised with MR and so its function has expanded beyond involvement in electrolyte transport to include regulation of corticosteroid action. Thus, 11β-HSD2 is highly expressed in fetal tissues, including the brain [28], and in the placenta where it is located at the interface between maternal and fetal circulations, in the syncytiotrophoblast in humans [29] and the labyrinthine zone in rodents [287]. This high expression of feto-placental 11β-HSD2 potentially serves as a ‘glucocorticoid barrier’ thus enabling tight regulation of materno-fetal glucocorticoid transfer.

## 11 11β-HSD2 deficiency: of mice and men

Hypertension associated with AME has been identified in approximately 100 cases worldwide [65]. The condition presents in childhood or young adulthood as severe hypertension, hypokalaemia, low renin and an extended half-life of cortisol as well as intrauterine growth retardation, short stature, thirst, polyuria and altered post-natal growth. Initially AME was attributed to elevated mineralocorticoid action but it was subsequently realised to be the consequence of defective cortisol metabolism, thus implicating impaired 11β-HSD2 activity [260,277]. Indeed, over 33 different mutations in 11β-HSD2, all autosomal recessive, have been identified and result in partial or total attenuation of enzyme activity [65].

An initial mouse model of targeted 11β-HSD2 disruption on an outbred background revealed mice with an apparently normal phenotype at birth but within 48 h, 50% exhibit motor deficiencies, perhaps due to hypokalaemia, and die [134]. Survivors are fertile, but exhibit severe hypertension, hypokalaemia and polyuria [134], all typical characteristics of AME and thus apparent mineralocorticoid actions of corticosterone were revealed by 11β-HSD2 deficiency. Interestingly, these mice did not exhibit reduced fetal weight although this was clearly apparent in later studies on a 11β-HSD2 knock-out model congenic on a C57BL/6 J background [101]. This raises the intriguing possibility of gene interaction effects on feto-placental 11β-HSD2 function.

In addition to replicating AME symptoms, 11β-HSD2^−/−^ mice revealed a key role for 11β-HSD2 in brain function. Heterozygous matings have shown 11β-HSD2^−/−^ offspring have heightened anxiety in comparison to their wild type littermates [101], demonstrating a key role of feto-placental 11β-HSD2 in prenatal glucocorticoid ‘programming’, a point which will be discussed in detail later.

## 12 Expression and action of 11β-HSD2 in the brain

### 12.1 11β-HSD2 in the adult brain

11β-HSD2 expression in the adult nervous system is low in comparison to classic MR target sites. Indeed, initial attempts at localising 11β-HSD2 expression in the brain failed [36,42,226]. However the development of sensitive mRNA probes coupled with extensive examination of the rat brain, localised moderate levels of 11β-HSD2 mRNA in scattered specific cells of the ventromedial and paraventricular (PVN) nuclei of the hypothalamus, amygdala, locus coeruleus, subcommissural organ and nucleus tractus solitarus (NTS) [224,227,322]. These areas underpin central control of blood pressure and sodium appetite, both features reported to be preferentially activated by aldosterone rather than corticosterone (reviewed in [79]) implying mediation by 11β-HSD2-protected MR. Most other MR-associated functions in the CNS are driven by glucocorticoids such as hippocampus-associated cognition [11,53,281], suggesting that the majority of MR-positive cells are 11β-HSD2 negative, reflecting their predominant occupancy by glucocorticoids *in vivo* [41,176,218].

Distribution of 11β-HSD2 within the mouse brain is limited even further, localised only to the NTS [104,105] which is consistent with a decreased aldosterone dependence on salt regulation in mice in comparison to rats [230]. This rather implies that any 11β-HSD2 mRNA outside the NTS-sodium appetite/central cardiovascular control circuitry is low-level expression without clear functional importance. In contrast, development of a transgenic mouse in which Cre recombinase was targeted to 11β-HSD2 suggested extensive distribution of 11β-HSD2 within the brain [192]. However, this most likely reflects the widespread expression of 11β-HSD2 in the developing brain driving developmental Cre expression which remains permanently activated thereafter.

Using real-time RT-PCR, 11β-HSD2 mRNA expression in the adult human brain has been reported in the amygdala, caudate nucleus, cerebellum, corpus callosum, hippocampus and thalamus [321], though the functional significance of low copy number transcripts is moot, especially in pooled samples of human post-mortem CNS when RNA preservation may be poor. Exploiting individual samples with very short-post-mortem delays (<4 h), no 11β-HSD2 mRNA or 11β-dehydrogenase activity was found in human cortex, hippocampus or cerebellum [233].

Moreover, 11β-HSD2 colocalisation with MR in the adult rodent brain is to date only clearly evident within the NTS, suggesting this is the major (perhaps only) locus with the potential for aldosterone-specific activation [79]. Curiously, adrenalectomized rats still exhibit c-Fos activation (an indicator of neuronal activation) in 11β-HSD2 neurons within the NTS after dietary sodium deprivation [75], which suggests that these 11β-HSD2 positive neurons are activated by factors additional to adrenal aldosterone and other corticosteroids. What these signals could be remains to be established but angiotensin II has been proposed [79]. Alternatively, the identification of 11-hydroxylase and aldosterone synthase activity (with encoding cyp11b1 and cyp11b2 mRNAs) within the brain points to local corticosteroid synthesis which may afford some impact in the absence of adrenal products [316]. 11β-HSD2 positive neurons within the NTS are innervated by the amygdala, PVN, dorsomedial NTS and components of the vagus [77,79,243,250], affording alternative pathways to activate the system. These inputs may act in conjunction with aldosterone to modulate NTS control of sodium appetite. It might be anticipated that NTS 11β-HSD2 neurons also project to areas involved in autonomic control of cardiovascular function. However to date, this does not seem to be the case and instead these axons innervate forebrain and forebrain-relay nuclei in the rostral brain stem [78] which associate with behavioural changes related to sodium appetite, reward, arousal and mood (reviewed in [79]).

The significance of 11β-HSD2 in other regions of the adult brain still remains elusive. Within the PVN, 11β-HSD2 mRNA has been detected using RT-PCR [76,224,227,322]. Whilst microinjection of either carbenoxolone or glycyrrhizic acid at high concentrations directly into the PVN increased sympathetic outflow and PVN activity [322] and these actions occurred via MR activation as the sympathoexcitory effects of carbenoxolone were blocked by intracerebroventricular spironolactone, an MR antagonist [322], these agents may have acted at different sites (i.e. PVN and NTS). Moreover, carbenoxolone and glycyrrhetinic acid also potently inhibit 11β-HSD1 which is also expressed in the PVN and other hypothalamic loci (at mRNA, immunoreactivity and enzyme activity levels; [180]) and may modulate glucocorticoid access to local MR as well as GR. So any functional role for 11β-HSD2 mRNA in the PVN remains uncertain. Moreover, the effects of functional expression of both isozymes in the same or even adjacent cells is uncertain, if and where this occurs. The notion of both destroying and regenerating glucocorticoids across the wall of the ER in a particular brain cell seems rather futile unless the balance is physiologically regulated somehow, a tough trick with two enzymes having such disparate affinities for substrate. The physiological importance of 11β-HSD2 in the forebrain requires clear functional definition. The various existing genetically-manipulated mouse and perhaps future rat models will be useful to dissect these questions in the absence of selective 11β-HSD2 inhibitors.

### 12.2 11β-HSD2. in the developing brain

In contrast to its limited expression in the adult CNS, 11β-HSD2 is highly expressed in the fetal brain where it appears critical for normal maturation and life-long function. The developing brain, as other fetal tissues, is extremely sensitive to glucocorticoids, which are crucial for normal cellular and biochemical maturation [132,167]. Thus glucocorticoids initiate terminal maturation, remodel axons and dendrites and determine programmed cell death [171]. In sheep, prenatal glucocorticoid administration retards brain weight at birth [107], delaying maturation of neurons, myelination, glia and vasculature [108]. The perinatal hippocampus is especially sensitive to glucocorticoids with consequences for subsequent memory and behaviour [25,247,259]. Thus, antenatal treatment of rhesus monkeys with dexamethasone causes dose-associated degeneration of hippocampal neurones and reduced hippocampal volume which persists at 20 months of age [279]. Prenatal stress (induced by repeated restraint of the pregnant female in the last week of pregnancy) reduces actively proliferating hippocampal cells and feminises sexually-dimorphic parameters of the adult hippocampus [161].

The critical nature of glucocorticoids for neural development is reflected by the expression of GR, MR and 11β-HSDs in the developing brain, with an intricate temporal and regional pattern [58,73,128]. In the embryonic rat brain, GR is highly expressed in neuroepithelium while MR expression is confined to the epithelium of the septal-hippocampal system, areas of the anterior hypothalamus, pituitary, deep layers of the superior colliculus, piriform cortex and lateral septum [58]. However, MR expression only becomes extremely prominent in the last 3 days of gestation within the hippocampus and lateral septum [58]. Interestingly, neural 11β-HSD2 expression does not coincide with the pattern of MR expression. Thus, 11β-HSD2 is abundant in neuroepithelium throughout midgestation and then strikingly and rapidly declines, coinciding with the terminal stage of neurogenesis in particular loci [28,58]. Similar patterns of expression occur in the human fetal brain with 11β-HSD2 silenced between gestational weeks 19–26 [261]. The lack of correspondence between MR and 11β-HSD2 expression patterns and the abundance of 11β-HSD2 in the fetal brain alongside GR supports the proposition that 11β-HSD2 acts to protect immature mitotically-active brain cells from premature exposure to the maturational effects of glucocorticoids. This is akin to the proposed role of placental 11β-HSD2 in protecting the fetus as a whole from overexposure to maternal glucocorticoids [28,242,287]. Abundant 11β-HSD2 expression in the developing CNS may act as an additional local barrier to endogenously-derived glucocorticoids. However, it still remains to be established if the presence of 11β-HSD2 in feto-placental tissues does indeed alter GR activation and occupancy or indeed target cell corticosteroid levels. Moreover, as discussed below, 11β-HSD2 may have further indirect neuroprotective effects.

The high levels of 11β-HSD2 observed during mid-term brain development reduce strikingly as brain areas cease to proliferate and differentiate. After birth, high levels of 11β-HSD2 are localised only in the proliferating external granular layer of the cerebellum and in several nuclei of the thalamus [224,227]. Therefore the cerebellum is sensitive in the early post-natal period to glucocorticoid-induced remodelling induced by either exogenous administration or in response to the stress induced by maternal separation [152,174,302]. Furthermore, cerebellar size is reduced in 11β-HSD2^−/−^ mice in early in post-natal life due to a decrease in the molecular and internal granule layers [104]. This associates with a delay in attainment of neurodevelopmental landmarks such as negative geotaxis and eye opening [104]. Thus, the timing of exposure of the developing brain to glucocorticoids seems to be tightly regulated by the presence of local 11β-HSD2 and the cell-specific patterns of its down-regulation during maturation.

## 13 Developmental regulation of 11β-HSD2

11β-HSD2 is expressed in the fetally-derived portion of the placenta, and regulated at the transcriptional, post-transcriptional and post-translational level by a host of factors including nitric oxide, progesterone, oestrogen, protein kinase A, retinoic acid, prostaglandins, catecholamines, oxygen, glucocorticoids, PPARΔ, proinflammatory cytokines and heavy metal toxins [7,92,93,122,133,204,207,236,270,271,275,280,310]. Furthermore, p38 MAPK has a specific role in upregulating 11β-HSD2 expression via alteration in 11β-HSD2 stability in primary trophoblast cells [246].

Within the brain, early weaning and social isolation decreases 11β-HSD2 expression in frontal cortex and hippocampus of piglets [211]. Recently, signalling by sonic hedgehog, a morphogen involved in the patterning of systems including the CNS, was shown potently to induce 11β-HSD2 in mouse cerebellar granule neuron precursors [95]. Whilst the mechanism(s) responsible for downregulating 11β-HSD2 in fetal brain at midgestation remain to be established, it may related to epigenetic silencing targeting the C and G-rich sequences in the 5′ region of the 11β-HSD2 gene [29]. Indeed, 11β-HSD2 expression is altered via epigenetic mechanisms in JEG-3 trophoblast cells [8].

## 14 The role of 11β-HSD2 in developmental programming

### 14.1 Developmental programming

A poor environment *in utero*, as for example indicated by low birth weight, can permanently alter the structure and function of organ systems, thereby increasing the offspring’s risk of cardiometabolic and neurobehavioural pathologies in later life. This notion of the developmental origins of adult health and disease is coined ‘developmental programming’. The environmental mechanisms of developmental programming have been ascribed to two major processes: fetal glucocorticoid exposure and fetal malnutrition. Glucocorticoids are crucial prenatally in the structural development and functional maturation of fetal organs. However, glucocorticoid overexposure of the fetus can be detrimental as glucocorticoids cause a shift from cell proliferation to differentiation. Therefore, exposure to excess glucocorticoids *in utero* alters fetal organ growth and maturation patterns, which can result in adverse consequences in later life. In humans, the actions of glucocorticoids are exploited for preterm births, serving to advance fetal lung maturation thereby reducing neonatal morbidity and mortality [222] although this may set the stage for adverse effects in later life [21,29,60–63,74,117,141,149,150].

The high expression of 11β-HSD2 in placenta and fetal tissues, the known gradient of glucocorticoids across the placenta, with cortisol levels in the maternal circulation ∼10-fold higher than in the fetus [19,47,183], and the growth retarding and maturational effects of glucocorticoids upon the fetus [171] have spawned the proposal that variations in feto-placental 11β-HSD2 may underlie developmental programming. In support, placental 11β-HSD2 correlates with birth parameters in rodents and, less consistently, in humans [21,190,262] suggesting that normal variation in fetal exposure to maternal glucocorticoids impact on fetal growth. Crucially, inhibition, deficiency or by-pass (poor substrate steroids such as dexamethasone or betamethasone) of 11β-HSD2 in gestation in rodents and humans associates with alterations in pregnancy duration, birth weight and programmed outcomes in the offspring [21,48,101,150,188,195,198,200,255,298,299,306,307]. Specifically, humans homozygous (or compound heterozygous) for deleterious mutations in *HSD11B2* have very low birth weight compared with their largely heterozygous siblings [48,188]. Similarly, 11β-HSD2^−/−^ mice have lower birth weight [101]. Furthermore, administration of dexamethasone or carbenoxolone reduces birth weight and exerts programming effects in rats [21,35,150,198,200,255,298,299,306,307]. In contrast, late pregnancy administration of metyrapone, an inhibitor of adrenal glucocorticoid synthesis, increases fetal and placental weight [35]. Mechanisms involving glucocorticoid-driven changes in target organ structure, gene expression and function have been demonstrated and epigenetic process maintaining such effects advocated [64,197,198,240,296,298,299,306,307].

Interestingly, in programming models involving maternal low-protein diet there is an increase in maternal and fetal glucocorticoid levels [89,148] in addition to a decrease in placental 11β-HSD2 activity [142]. Moreover, dexamethasone administration during pregnancy decreases food intake [305]. Consequently, there seems to be considerable overlap in mechanisms by which maternal undernutrition and fetal glucocorticoid overexposure elicit developmental programming.

The significance of fetal glucocorticoid exposure for adult pathophysiology has been studied in detail in the rodent, in particular the rat, but studies have found similar processes in the guinea pig and sheep [21,29,60–63,74,117,141,149,150]. Whilst all such models show that a variety of maternal insults exert remarkably similar, though not identical, effects upon offspring physiology, extrapolation to humans has remained unresolved, not least because of species differences in placental anatomy and the detailed ontogeny of 11β-HSD2 expression [28,266]. Crucially, recent work in singleton-bearing non-human primates has shown that exposure in late gestation to dexamethasone, a synthetic glucocorticoid which is a poor substrate for inactivation by 11β-HSD2, causes adverse cardiometabolic and neuroendocrine sequellae in the juvenile offspring [55].

### 14.2 Modulation of neural 11β-HSD2 impacts on neural development and subsequent adult function

In the rat, central programming by glucocorticoids, be it from maternal administration of dexamethasone or prenatal stress produces offspring that appear more anxious as adults. Thus, late gestational dexamethasone exposure in rats impairs the offspring’s ‘coping’ behaviours in aversive situations later in life as exemplified by reduced exploration in the open field test and elevated-plus maze [299]. Such increase in anxiety-like behaviour is evident as early as post-natal week 10 in rats prenatally exposed to dexamethasone [191]. Moreover, 11β-HSD2 appears important in these events since either treatment of pregnant rats with an 11β-HSD inhibitor or gene deletion in mice produces offspring with enhanced anxiety-related behaviours [101,298]. However, one must remember that in the 11β-HSD2^−/−^ mouse there is no 11β-HSD1 substrate (11-dehydrocorticosterone) and therefore this will have ramifications for brain function, albeit perhaps most notably with ageing.

These programmed changes in behaviour are accompanied by alterations in the HPA axis. Thus, maternal dexamethasone treatment increases corticosterone and ACTH levels in the adult offspring, although interestingly, mostly in males [149,189,200,299]. These effects seem to reflect a change in the feedback of the HPA axis at the level of the hypothalamus, since CRH mRNA increased in the paraventricular nucleus whereas hippocampal MR and GR both decreased [46,298]. Furthermore, the HPA axis period of hyporesponsiveness in early post-natal life is abolished in adult rats exposed to prenatal stress [156], whilst normal age-related HPA-axis dysfunction is accelerated by prenatal stress [202]. In sheep, a single injection of betamethasone on gestational day 104 altered HPA function in offspring at 1 year of age, with elevated basal and stimulated plasma cortisol concentrations [253]. In contrast, repeated maternal betamethasone injections elevated the ACTH responses in the offspring to a CRH/AVP challenge in addition to increased basal ACTH levels but decreased basal and stimulated cortisol levels [187,253]. In primates, offspring of mothers treated with dexamethasone during late pregnancy have elevated basal and stress-stimulated cortisol levels [55,278].

Moreover, prenatal stress and alterations in offspring HPA axis function has also been associated in humans. Thus, children of mothers present at or near to the World Trade Centre atrocity on 9/11, who themselves developed symptoms of post-traumatic stress disorder (PTSD), had lower cortisol levels [318]. Importantly these changes were most apparent in babies born to mothers who were in the last three months of their pregnancies when the trauma occurred, suggesting these observations can be attributed to developmental programming phenomena as opposed to a genetic susceptibility or the presence of PTSD *per se* [318]. Such effects may transmit into subsequent generations, since healthy adult children of Holocaust survivors with PTSD (and therefore lower plasma cortisol levels) themselves have lower cortisol levels though no PTSD [319]. This appears to be confined to the children of Holocaust-exposed mothers with PTSD [319]. In contrast to PTSD, maternal anxiety and depression seem to elevate cortisol in the child [199,282]. Therefore the mechanisms of prenatal stress programming HPA function in humans seem complex, with possibly different pathways involved. Intriguingly, in Finland, women who voluntarily ingest liquorice-containing foodstuffs (that potently inhibit placental 11β-HSD2 [20]) in pregnancy have somewhat shorter gestations and their 8-year old offspring show altered cognitive function, affective disturbances (notably markedly increased rates of attention-deficit hyperactivity disorder), HPA axis hyperactivity and sleep disturbances [212,213].

Behavioural changes in adults exposed prenatally to glucocorticoids appear associated with altered functioning of the amygdala, the key structure involved in the expression of fear and anxiety, with amygdala CRH levels implicated in fear-related behaviours. Prenatal glucocorticoid exposure increases adult CRH levels specifically in the central nucleus of the amygdala and therefore may be responsible for the increase in anxiety-like behaviour observed in these animals. Prenatal stress similarly programmes increased anxiety-related behaviours with elevated CRH in the amygdala [46] as well as schizophrenic-like behaviour [130,147] which can be reversed by administration of oxytocin into the central amygdala [147]. Moreover, corticosteroids facilitate CRH mRNA expression in this nucleus [106] and increase GR and/or MR in the amygdala [298,299]. A direct relationship between brain corticosteroid receptor levels and anxiety-like behaviour is supported by the phenotype of transgenic mice with selective loss of GR gene expression in the brain, which shows markedly reduced anxiety [276]. Furthermore, in human depression and schizophrenia, decreases in GR expression in specific brain regions such as the amygdala and hippocampus have been reported [208,297]. Interestingly, forebrain-specific knock-out of GR results in mice with increased depressive-like behaviour and reduced anxiety-related behaviour [24], whilst forebrain-specific MR-overexpressing transgenic mice exhibit reduced anxiety and altered behavioural response to novelty [136]. It is unclear however, how depression and anxiety relate and whether they represent different disorders or have similar underlying dysfunction. Regardless, alterations in brain GR and MR appear to be driving forces behind the anxious phenotype.

**Fig. 6:**
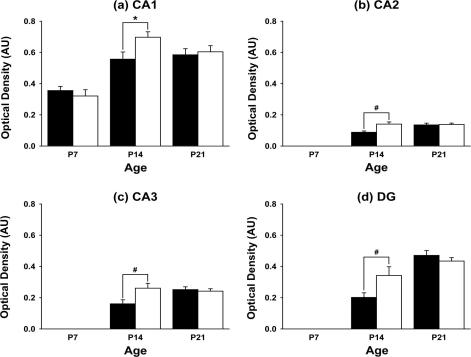
Hippocampal GR mRNA expression during early post-natal period in 11β-HSD2 transgenic mice. Effect of 11β-HSD2 genotype (+/+ ■, ^−/−^ □) on glucocorticoid receptor (GR) mRNA expression in the (a) CA1, (b) CA2, (c) CA3 and (d) dentate gyrus (DG) hippocampal subfields. Expression levels were measured in 1-week (P7), 2-week (P14) and 3-week (P21) old mice by optical densitometry of *in situ* hybridisation autoradiographs. Data are expressed as mean ± SEM in arbitary units (AU) and analysed by two-way ANOVA with SNK *post-hoc* testing for each subfield; *n* = 5–10 per group. ^*^P < 0.05, ^#^*P* < 0.01.

Interestingly, despite increased anxiety, the HPA axis activity of 11β-HSD2^−/−^ offspring appears unaffected, perhaps a reflection of the additional effects of attenuated HPA axis reactivity due to reduced glucocorticoid clearance with absence of renal 11β-HSD2 [101]. However, as predicted, adrenal size is reduced and hence resetting of the HPA axis may have occurred during development. This, together with decreased degradation of corticosterone, means less corticosterone needs to be produced. Consistent with this, 11β-HSD2^−/−^ mice exhibit no differences during adulthood in the limbic expression of GR, MR or CRH, but there are transient changes within the post-natal period. In homozygous matings of 11β-HSD2^−/−^ mice, transient elevations in GR transcript were observed by *in situ* in all hippocampal subfields of 11β-HSD2^−/−^ offspring at P14 (Fig. 6; C.T. Abrahamsen, M.C. Holmes, unpublished observations). Similar transient changes were observed with MR, Sgk1, Fkbp5 and BDNF (C.T. Abrahamsen, M.C. Holmes, unpublished observations). Interestingly, preliminary data suggests that altered serotonin signalling in 11β-HSD2^−/−^ adult brains may be responsible, at least in part, for the anxiety-related behaviour (C.S. Wyrwoll, M.C. Holmes, unpublished observations).

### 14.3 The placenta: an indirect role for 11β-HSD2 in neuroprotection

**Fig. 7:**
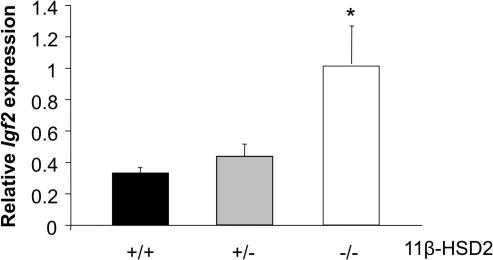
Total Igf2 expression in liver of 11β-HSD2^+/+^, 11β-HSD2^+/-^ and 11β-HSD2^−/−^ fetuses at E15. Total Igf2 expression was measured by real-time RT-PCR in offspring generated from heterozygous 11β-HSD2 matings. Data are expressed as mean ± SEM; *n* = 6 per group. ^*^P < 0.05.

As outlined above, it has been hypothesised that relative deficiency of placental 11β-HSD2 may underpin aspects of developmental programming by allowing excess glucocorticoid passage from the ‘high’ glucocorticoid maternal circulation to the ‘low’ glucocorticoid fetal environment [67]. The observed impairment of fetal growth in these studies is frequently attributed to direct effects of glucocorticoids on the fetus. Fetal growth is however dependent on an array of maternal, placental and fetal endocrine signals and glucocorticoid-mediated fetal growth retardation must also relate, at least in part, to disturbances in placental growth and function. Indeed, maternal treatment with dexamethasone impairs normal vascular growth in the rat placenta [100]. Moreover, in addition to impaired vascularity, placentas from 11β-HSD2^−/−^ fetuses exhibit altered placental transport of glucose and amino acids [308]. Thus, amino acid transport in placentas from 11β-HSD2^−/−^ fetuses is up-regulated at E15 which coincides with maintained fetal weight but by E18, fetal 11β-HSD2^−/−^ weight is decreased alongside reduced placental glucose transport [308]. Interestingly, at E15, brain and liver corticosterone levels are only slightly higher in 11β-HSD2^−/−^ fetuses (C.S. Wyrwoll, M.C. Holmes, unpublished observations), suggestive of an additional placental glucocorticoid ‘barrier’ although what this could be remains moot; mdr1/p-glycoprotein has been advocated [123,162]. Extensive further work is required to establish whether placental transfer of other factors such as essential fatty acids and oxygen contribute to altered fetal development and to elucidate the involvement of factors within the placenta such as VEGF and IGFs in altering placental function. Indeed, Igf2 expression is up-regulated in the livers of 11β-HSD2^−/−^ fetuses at E15 (Fig. 7; A. Reddy, C.S. Wyrwoll, M.C. Holmes, unpublished observations). The significance of this expression is uncertain but it may be an indicator of crosstalk between the fetus and placenta [43]. Nonetheless, the current data provide a convincing argument that while maternal glucocorticoids could play a direct role in programming the fetus, notably its brain, placental development and function plays a key role. It must be noted however, that until tissue specific knockouts of 11β-HSD2 in placenta and fetal tissues, in particular the brain, are developed, the differential significance of feto-placental 11β-HSD2 for development cannot be elucidated.

## 15 Overview

**Fig. 8:**
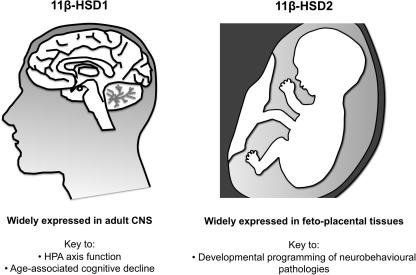
Expression and function of 11β-hydroxysteroid dehydrogenase (11β-HSD) type 1 and 2. 11β-HSD1 is widely expressed throughout the adult CNS and is key to HPA axis function and cognitive decline during ageing. Conversely, the major central effects of 11β-HSD2 are seen in development, as expression of 11β-HSD2 is high in fetal tissues including the neonate brain and placenta. Loss of 11β-HSD2 from the fetus and fetally-derived tissues results in a life-long phenotype of anxiety, consistent with developmental programming.

The past decade has seen considerable progress in the understanding of the role of 11β-HSDs in neural function, particularly aided by the development of transgenic animals. 11β-HSD1 plays myriad roles in normal function of the adult brain (Fig. 8), with wide distribution throughout the CNS, and doubtless more roles will emerge. Furthermore, 11β-HSD1 appears critical to ageing brain function, with age-related increases in 11β-HSD1 linked to a decline in cognition. This opens up potential therapeutic avenues, setting the stage for the development of selective 11β-HSD1 inhibitors for cognitive decline to build upon intriguing effects in null mice and with non-selective inhibitors in animals and humans. With regards to 11β-HSD2, its significance within the adult brain at present seems confined to controlling salt appetite, presumably in salt-seeking species, but this isozyme plays a crucial role during development (Fig. 8). Both fetal neural and placental 11β-HSD2 appear to be a central hub for eliciting the programmed effects on neuropsychiatry, although the relative significance of fetal brain vs. placental 11β-HSD2 is yet to be established. It is anticipated that the next decade will see exploitation of this understanding to generate human impacts, such as prediction (measuring placental 11β-HSD2 levels or its epigenetic marks) and manipulation of developmental programming on the risk of CNS disorders and the use of emerging selective 11β-HSD1 inhibitors in disorders of the ageing brain.
